# Association between Intestinal Parasite Infections and Proxies for Body Composition: A Scoping Review

**DOI:** 10.3390/nu14112229

**Published:** 2022-05-26

**Authors:** Idalécia Cossa-Moiane, Clémentine Roucher, Tamlyn Mac Quene, Maiza Campos-Ponce, Nilsa de Deus, Katja Polman, Colleen Doak

**Affiliations:** 1 Instituto Nacional de Saúde (INS), Maputo 264, Mozambique; nilsa.dedeus@ins.gov.mz; 2 Department of Biomedical Sciences, Institute of Tropical Medicine, 2000 Antwerpen, Belgium; clementine.roucher@live.fr (C.R.); kpolman@itg.be (K.P.); 3 Centre for Global Surgery, Department of Global Health, Faculty of Medicine and Health Sciences, Stellenbosch University, Matieland 7602, South Africa; tamlyn.macquene@gmail.com; 4 Faculty of Science, Vrije Universiteit Amsterdam, 1081 HV Amsterdam, The Netherlands; m.camposponce@vu.nl; 5 Departamento de Ciências Biológicas, Universidade Eduardo Mondlane, Maputo 3453, Mozambique; 6 Center for Health Sciences Education, College of Health Sciences, St. Ambrose University, Davenport, IA 52803, USA; doakcolleenm@sau.edu

**Keywords:** intestinal parasite infections, body composition, underweight, body fat, association

## Abstract

It has long been recognized that intestinal parasite infections and undernutrition are closely linked. However, little is known about the role of intestinal parasite infections (IPIs), or parasite clearance, in these processes. The aim of this scoping review was to summarize published evidence on the association between IPIs and body composition. PRISMA guidelines, PubMed/MEDLINE, EMBASE and Cochrane Library databases were searched up until June 2021. Studies reporting on IPIs in relation to (proxies for) body composition were eligible. Study quality and risk of bias were assessed using Joanna Briggs Institute (JBI) critical appraisal tools. Twenty-four studies were included, two Randomized Control Trials (RCTs) and 22 observational. Most observational studies showed IPIs to be associated with lower Body Mass Index (BMI) or being underweight as proxies for body composition. One RCT showed no effect of antiparasitic treatment on body composition, while the other one showed a significant post-treatment increase in body fat, as measured by BMI z-scores and skinfolds. This review lends support to distinct associations between IPIs and body composition. More longitudinal studies are needed using direct measures of body composition to investigate whether weight gained after antiparasitic treatment concerns an increase in body fat or healthy weight gain.

## 1. Introduction

Intestinal parasite infections and undernutrition are major health problems in low- and middle-income countries (LMIC). According to World Health Organization (WHO), more than 1.5 billion people, or 24% of the world’s population, are infected with soil-transmitted helminths, the largest group of intestinal parasite infections, contributing 5.2 million disability-adjusted life-years worldwide in 2010 [1,2]. Globally, 47 million children under 5 years of age are wasted, and 144 million are stunted [3]. Around 45% of deaths among children under 5 years of age are linked to undernutrition [3].

Prior research has demonstrated that undernutrition early in life is associated with an increased risk of a number of chronic disease outcomes [4,5,6,7,8,9]. Further studies have shown that the increased risk of chronic disease is specifically related to accelerated weight gain [10] even during recovery from undernutrition [11,12]. Research from Brazil has shown that stunted children deposited more fat and suffered more from overweight and obesity than non-stunted children [13]. Furthermore, undernourished children were likely to experience rapid weight gain during nutritional rehabilitation [14]. Moreover, studies have shown that during a period of rapid weight gain, fat mass is disproportionately accrued over fat-free mass [15]. Hence, undernutrition and nutritional recovery are associated with weight gain and body fat deposits.

It is well-known that intestinal parasite infections and early child undernutrition are closely interrelated [14,16,17]. However, little is known about the relationship between intestinal parasite infections, parasite clearance, and weight gain or (changes in) body fat. Sawaya et al. [8] showed that a combination of dietary and anti-parasitic measures was able to alter metabolic changes in Brazilian children. Moreover, a study in Chile suggested that dietary interventions which only target undernutrition can lead to an increased risk of overweight/obesity [18]. In addition, Mexican children infected with *Entamoeba coli* were found to have a significantly higher waist circumference, waist to height ratio, and percentage of body and abdominal fat than children not infected or with a light infection [19]. These results suggest that intestinal parasite infection may play a role in the relationship between child undernutrition and overweight/obesity [20].

The importance of clarifying the relationship between intestinal parasite infection, early child undernutrition, weight gain and body fat deposits is underscored by documented global trends of increasing overweight and obesity prevalence [21] in LMIC. Current estimates show that 3.3 billion adults will be overweight or obese by 2030 [20]. This rapid rise in overweight and obesity is of particular concern because it occurs in addition to continuing child undernutrition [22], and in countries where parasitic infections remain endemic. In this context, the aim of this review is to summarize published findings reporting on the association between intestinal parasite infections (IPIs) and body composition.

## 2. Materials and Methods

### 2.1. Data Sources and Search Strategy

Searches were conducted on MEDLINE, EMBASE and Cochrane Library. Search terms included several variations on body fat, BMI, body composition, overweight, obesity, parasitic infections, parasites, helminths, intestinal parasites, protozoa, *Entamoeba coli*, *Giardia*, *Entamoeba histolytica*, *Cryptosporidium*, *Ascaris*, *Trichuris*, *Ancylostoma*, *Necator*, hookworm, whipworm, roundworm, pinworm, deworm, *Strongyloides* in the titles and abstracts of studies and where possible as MeSH terms or Emtree terms (Table 1). The references obtained from the three search engines were merged in a single file using Endnote X8-software (Thompson Reuters, San Francisco, CA, USA). After removing duplicates, the titles, abstracts, and keywords were reviewed for inclusion. When the articles met the inclusion criteria, they were entirely scrutinized and the data were extracted.

A first search was conducted in June 2021 by ICM and replicated by a second researcher in August 2021 by CR. The review was carried out in accordance with the PRISMA reporting checklist [23] for scoping reviews (Table S1).

### 2.2. Eligibility Criteria

#### 2.2.1. Study Inclusion Criteria

No restrictions by study design were applied. Thus, all published, relevant, peer-reviewed scientific articles including randomized controlled trials (RCTs), other interventions, or observational studies were eligible for inclusion. Studies were included if they reported on intestinal, i.e., protozoan or helminthic, parasitic infections and one or more measures of body composition. Studies that used microscopy, molecular and/or serological tests to detect intestinal parasite infection were selected; other methods of assessment (e.g., questionnaires) were not eligible. Studies were eligible if they reported on body composition using measures that distinguish between fat and fat-free mass, such as skinfolds, Dual-energy X-ray absorptiometry—DEXA/DXA, and bio-electrical impedance analysis (BIA). In addition, studies were selected if they reported proxy measurements of body composition using weight-for-height measures, such as body mass index (BMI), BMI z-scores (BMIZ), and weight-for-height z-scores (WHZ), or overweight/obesity using WHO definitions [24]. Studies with any effect estimates or statistical comparisons indicating associations between intestinal parasite infection and body composition were included, such as mean values/differences (±Standard Deviations, SD’s), Odds Ratios (OR) and Relative Risk (RR) with 95% Confidence Intervals (CI), *p*-values (*p* < 0.05 significance) or regression coefficients.

#### 2.2.2. Study Exclusion Criteria

Studies were excluded if they were not in English, were non-human studies and/or were not published in peer review journals. Additionally, all review articles, editorial papers and abstracts from symposia, conferences, or seminars were excluded. Articles reporting on interim results or incomplete findings did not meet the eligibility criteria. All published studies related to parasites other than intestinal parasites were excluded. Similarly, studies reporting only anthropometric measures, such as height or stunting, but not on body composition were excluded. Lastly, studies that reported having measured intestinal parasite infections and body composition but did not specifically analyze or report on the association between the two were also excluded.

### 2.3. Quality Assessment of Included Studies

The quality of eligible studies and the potential for risk of bias were independently assessed by two reviewers; any discrepancies were resolved by discussion or arbitration by a third reviewer. The risk of bias was assessed by using the Joanna Briggs Institute (JBI) critical appraisal tool [25]. The checklists for cross-sectional and RCTs studies were applied with 8 and 13 questions, respectively. Each study was scored according to the structured questions. The responses were scored 0 for “Not reported”, 1 for “Yes” and NA for “Not Applicable”. Studies with medium (fulfilling 50% of quality assessment parameters) and high quality were included for analysis.

### 2.4. Data Extraction and Comparison

Extracted data included first author/year, study setting and design, the number of participants, age, intestinal parasites identified, and body composition measures used. Moreover, the time frame of the observational studies was included.

From studies identified as RCTs, additional information pertaining to antiparasitic treatment and follow-up times was also included. Observational studies were described and compared separately from RCTs. For the observational studies, results could only be compared between studies using similar measures of anthropometric outcomes. Where mean differences were not reported, these were calculated using the group means reported by the authors. All other results were reported as presented by the authors.

## 3. Results

**Figure 1:**
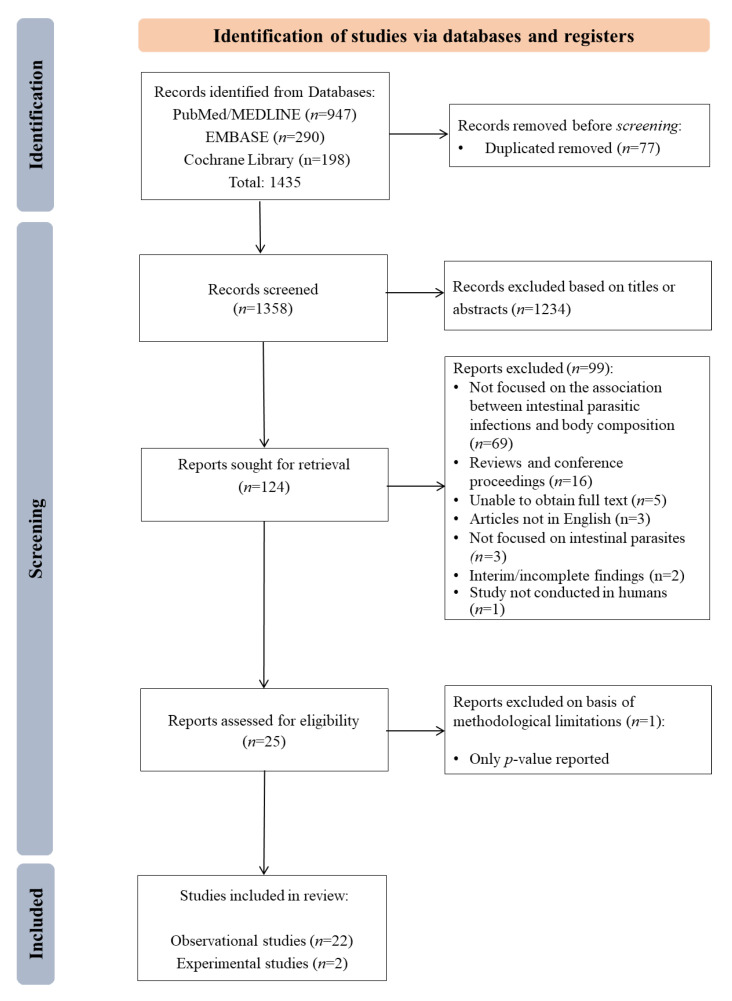
Flowchart of the search and selection of the studies for review.

The results of combined searches from MEDLINE, EMBASE and the COCHRANE Library databases are shown in Figure 1. From the three search engines, 1435 references were obtained. Duplicate references were identified and removed, leaving 1358 references. After screening, 124 articles for full-text screening remained. Based on the potentially relevant full-text articles, 25 articles met the initial criteria for inclusion in this review. We further excluded one article only reporting a *p*-value with no indication of the effect estimate or the direction of the association and another one that did not compare if weight categories (by BMI) were related to helminth/no helminth infection, leaving 24 articles, of which 22 reported on observational studies and two on experimental studies (randomized controlled trials). All the studies were included based on the JBI quality assessment criteria as they all fulfilled 100% of the quality assessment parameters (Table S2).

The characteristics of all studies included in this review are presented in Table 2. Studies were carried out worldwide, with a majority of studies being from South America and Asia. Most study designs were reported as cross-sectional or retrospective, with only two of the observational studies being reported as longitudinal. The sample size of the studies varied, ranging from smaller studies (*n* = 150) to larger studies (*n* = 16,347). Although most studies were conducted on children, the age of study participants varied from infants to the elderly. Most of the intestinal parasite infections measured were soil-transmitted helminths (i.e., *Ascaris*, *Trichuris* and hookworm) and BMI was the basis for most of the reported body composition measures.

**Table 2:** Characteristics of the studies included.

Author, Year	Study Setting, Years	Study Design	No. of Participant	Age of the Participants	Intestinal Parasite Infections (IPI’s) Reported	Body Composition Reported	References
Amare et al., 2013	Ethiopia, 2008	Cross-Sectional	405	12.09 ± 2.54 y	*Ascaris,* Hookworm, *Trichuris*, *Strongyloides Giardia*, *Entamoeba* spp.	BMI, BMIZ	[26]
Campos-Ponce et al., 2012	Venezuela, 2010	Cross-Sectional	225	0–19 y, >19 y	*Giardia*, Geohelminths, IPI	BMI	[18]
Casapia et al., 2007	Peru, 2005–2006	Cross-Sectional	252	<5 y	*Ascaris*, Hookworm, *Trichuris*	WHZ	[27]
Chu et al., 2013	Taiwan, 2010	Cross-Sectional	11,080	7–14 y	Pinworm	BMI	[28]
Geltman et al., 2001	USA, 1995–1998	Cross-Sectional	1825	<18 y	IPI	WHZ, BMI	[29]
Gerber et al., 2018	South Africa, 2015	Cross-Sectional	801	8–12 y	*Ascaris*, *Trichuris*	BMI, Body fat	[30]
Jardim-Botelho et al., 2008	Brazil, 2004	Cross-Sectional	1113	0–18, >18 y	*Ascaris*, Hookworm, *Trichuris, Strongyloides*	Triceps SF, WHZ, BMI, BMI/AGE, MUAC	[31]
Kurscheid et al., 2020	Indonesia, 2015	Cross-Sectional	998	2–12 y	*Ascaris*, Hookworm, *Trichuris*	BMI	[32]
Lander et al., 2015	Brazil, 2010	Cross-Sectional	376	3–6 y	Helminths	WHZ, BMI, BMIZ	[33]
Li et al., 2015	China, 2012	Cross-Sectional	369	50–70 y	Hookworm, *Ascaris*, Protozoa, Helminths	BMI	[34]
Orden et al., 2014	Argentina, 2009–2011	Cross-Sectional	708	3–11 y	*Giardia*, *Ascaris*, *Trichuris*	BMI, Triceps, Subscapular SF, BMIZ	[35]
Patel and Khandekar, 2006	Oman, 2004–2005	Cross-Sectional	436	9–10 y	*Entamoeba*, *Giardia*, *Ascaris*, *Trichuris*, Hookworm, *Strongyloides*	BMI	[36]
Rivero et al., 2018	Argentina, 2017	Cross-Sectional	303	<15 y	Helminths (*Enterobius*, Hookworms, *Strongyloides*, *Hymenolepis*, *Ascaris*), Protozoans (*Giardia*, *Entamoeba*, *Blastocystis*, *Chilomastix*, *Endolimax*, *Cryptosporidium*, *Iodamoeba*)	BMIZ	[37]
Quihui-Cota et al., 2015	Mexico, 2008–2009	Cross-Sectional	405	6–13 y	*Cryptosporidium*	BMI/AGE	[38]
Sanchez et al., 2013	Honduras, 2011	Cross-Sectional	320	7–14 y	*Ascaris*, Hookworm, *Trichuris*, STH	BMI/AGE	[39]
Sayasone et al., 2015	Lao PDR, 2007	Cross-Sectional	1313	6 mo–12 y	*Ascaris*, Hookworm, *Trichuris*	BMI/AGE, WHZ	[40]
Stephenson et al., 1989	Kenya, 1986	Randomized controlled trial	150	6–16 y	*Ascaris*, Hookworm, *Trichuris*	WH, Triceps, subscapular SF	[41]
Verhagen et al., 2013	Venezuela, 2010–2011	Cross-Sectional	390	4–6 y	*Ascaris*, *Trichuris*, Hookworm, *Giardia*, *Strongyloides*, Helminths	BMI/AGE, WHZ	[42]
Wiria et al., 2013a	Indonesia, 2008–2010	Randomized controlled trial	4004	>2 y (at semi-urban areas) and 5–15 y (at rural areas)	Helminths, *Ascaris*, Hookworm, *Trichuris*, *Strongyloides*, *Ancylostoma*	BMI	[43]
Wiria et al., 2013b	Indonesia, 2009	Cross-Sectional	675	18–80 y	*Ascaris*, *Trichuris*, *Strongyloides*, *Ancylostoma*	BMI	[44]
Zavala et al., 2016	Mexico, 2013	Cross-Sectional	296	6–10 y	*Ascaris*, *Balantidium coli*, Hookworm, *Giardia*, *E. coli*, *E. histolytica*, *E. nana*,	BMI/AGE, Body Composition (Body Fat)	[19]
Zavala et al., 2019	Mexico, 2000, 2006 and 2012	Ecological	1–5 y (8927), 6–10 y (16,347) and 11–19 y (13,992)	1–19 y	*Ascaris*, Protozoa	BMI/AGE	[38]
Zhou et al., 2007	China, 2001–2005	Longitudinal	274	10–12 y	*Ascaris*, *Trichuris*	BMI, BMI/AGE	[34]

BMI: Body mass index; BMI/AGE: BMI-for-age z-score; BMIZ: Body mass index z-score; mo: months; MUAC: Mid-Upper Arm Circumference; NR: Not reported; SF: skinfolds; y: years old; WH: Weight-for-height; WHZ: Weight-for-height z-score.

Table 3 shows the results of the experimental (RCT) studies. Stephenson et al. [17] reported that albendazole treatment was associated with a greater increase in both triceps and subscapular skinfolds and a greater increase in weight-for-height percentage compared to the placebo group at 6 months follow-up. Wiria et al. [43] found no significant change in BMIZ or BMI, in children and adults, respectively, at 9 and 21 months after treatment.

The results of observational studies reporting on the association between intestinal parasite infections and direct measures of body composition are presented in Table 4. Zavala et al. [19] showed that children infected with intestinal parasites tended to have higher body fat (in kg) but less abdominal or body fat (in %) but these results were not statistically significant. In the same article, Zavala et al. [19] described a species-specific effect of the non-pathogenic intestinal parasite *Entamoeba coli* (results not shown in Table 4). According to them, children with a moderate-heavy infection with *E. coli* had significantly higher body fat and abdominal fat compared to children that were not infected or had a light intensity infection (*p* < 0.05). Similar results were observed in moderate-heavy infection with *E. nana* and in light infection with *Ascaris;* however, the results were not statistically significant. The results of Jardim-Botelho et al. [31] showed that both children and adults that were infected with either *Ascaris* or hookworm were more likely to have lower body (fat and fat-free) mass using skinfold measures. Gerber et al. [30] showed that for every unit increase in skinfolds, children were less likely to be infected by *Ascaris*, *Trichuris,* or both, although these results were not statistically significant.

Table 5 shows the results of the observational studies that describe the association between intestinal parasite infection status and indirect measures/proxies of body composition, reported mainly as BMI followed by WHZ and dichotomous evaluations of weight-for-height measures. Both Wiria et al. [44] and Li et al. [34] showed that adults infected with intestinal parasites were more likely to have a lower BMI (*p* < 0.05). This same pattern was observed in children using various measures of weight-for-height, as reported by Gerber et al. [30], Verhagen et al. [42], Zhou et al. [45], Geltman et al. [29], Chu et al. [28], Sayasone et al. [40], Lander et al. [33] and Zavala et al. for *Ascaris* infection (*p* < 0.05) [46]. Several other studies also showed parasitic infections associated with lower weight-for-height indicators, although the outcomes were not statistically significant (Patel and Khandekar [36], Casapia et al. [27], Amare et al. [26], Sanchez et al. [39], Orden et al. [35] and Rivero et al. [37]). Zavala et al. showed an opposite, statistically significant association for intestinal protozoa [46] and for IPI [19]. The same pattern was also found by Quihui-Cota et al. [38] and Campos Ponce et al. [18], although it was not statistically significant. Wasilewska et al. [47] found no association between BMIZ and *Ascaris* infection in children (OR = 0.98). Likewise, Kurscheid et al. [32] found no association between BMI and *Trichuris* and/or hookworm and/or *Ascaris* infection. However, the results of both studies were not statistically significant.

## 4. Discussion

We identified 24 studies that reported on the association between intestinal parasite infection and body composition. Of these, most used weight-for-height measures (BMI, BMIZ or WHZ) as proxies for body composition. The observational studies showed a clear and consistent pattern by which intestinal parasite infection was associated with a lower BMI or with being underweight. Likewise, those with intestinal parasites were less likely to be overweight/obese. These findings are consistent with the existing literature showing parasitic infection to be associated with undernutrition, such as stunting or micronutrient deficiency [48,49].

Consistent with the findings of the observational studies, the RCT by Stephenson et al. [17] found albendazole treatment to be associated with a greater increase in weight-for-height percentage than the comparison group, a result that was confirmed using skinfold measures as a more direct measure for body fat. The potential increase in weight is even more important given the findings of a systematic review by Welch et al. [50] showing deworming to have no effect on growth in height. In contrast, the RCT by Wiria et al. [43] showed almost no change in BMI after albendazole treatment. However, this study was carried out in a broader age group, including adults. Children are more vulnerable to adipose weight gain than adults because the relationship between adiposity development and rapid weight gain is thought to be driven by growth hormones [51]. These RCTs used BMI and skinfolds considering that they are simple, inexpensive and easily applicable in fieldwork. Moreover, the tools suggested in each study are ideal considering the age of the participant but less sensitive to directly determining body composition.

It is important to note a number of limitations in this review. Since the studies used different (in)direct measures of body composition (BMI, BMIZ, WHZ, Mid-Upper Arm Circumference—MUAC, skinfolds taken at different time points, and DEXA), results could not be directly compared, and therefore, a meta-analysis could not be done. The limitations of weight-for-height measures (BMI/BMIZ/WHZ) of body composition are well known [52]. Although weight-for-height measures are commonly used as indicators of thinness, wasting, underweight, overweight and obesity, these measures do not distinguish between weight gained as fat versus fat-free mass [50]. Some studies reported skinfold measures, which were taken in different places, such as triceps or subscapular skinfolds. Skinfold measures provide more direct proxies for body fat and fat distribution than weight-for-height measures, but the validity is sensitive to the skills of the anthropometrist [50].

In addition to different outcomes related to body composition, the studies used different age groups, with some studies specifically looking at a narrow age range in children, others with more broad age ranges only in adults or only in children, and others combining both adults and children. Moreover, as can be seen from Table 2, nearly every study investigated different (sets of) parasitic infections, and only a few reported findings separately by parasite species. Additionally, the respective studies showed important variations by country and year. These (and other) differences in methodology, design and context hampered direct comparison between studies and may explain some of the inconsistent findings, in particular for the two RCT studies.

Despite the differences, there is consistency across multiple studies lending support to the association between intestinal parasite infections and body composition. As previously stated, we do not know whether the thinness related to parasite infections is due to loss of fat-free mass, loss of fat, or both together. The same holds for weight gain related to parasite clearance and whether this can be attributed to muscle gain, fat gain, or both. However, the findings by Stephenson et al. [17] imply that, under some circumstances, antiparasitic treatment can be related to a relative increase in fat mass.

## 5. Conclusions

While the treatment of parasitic infections is generally assumed to be essential to recovery from undernutrition, it is important to study changes in body composition to ensure healthy weight gain after recovery from parasitic infection. Longitudinal studies are needed to monitor individual, household and community risk factors that can contribute to changes in body composition after antiparasitic treatment. Such studies are of particular interest in Africa, where intestinal parasite infections are still endemic and where obesity is likely to emerge and/or spread. Additionally, these studies should target body composition using more sensitive tools, such as computed tomography (CT) scanning, or DEXA/DXA and/or BIA scanning.

## Figures and Tables

**Table 1:** Search strategies used in the Databases.

Database	Search Terms
MEDLINE	#1 ((((body fat) OR (BMI)) OR (body composition)) OR (overweight)) OR (obesity)
	#2 ((((((((((((((((((parasitic infections) OR (parasites)) OR (helminth)) OR (intestinal parasite)) OR (protozoa)) OR (*Entamoeba coli*)) OR (*Giardia*)) OR (*Entamoeba histolytica*)) OR (*Cryptosporidium*)) OR (*Ascaris*)) OR (*Trichuris*)) OR (*Ancylostoma*)) OR (*Necator*)) OR (hookworm)) OR (whipworm)) OR (roundworm)) OR (pinworm)) OR (deworm)) OR (*Strongyloides*)
	#3 Humans
	#4 (combination)—((((((body fat) OR (BMI)) OR (body composition)) OR (overweight)) OR (obesity) AND (((((((((((((((((((parasitic infections) OR (parasites)) OR (helminth)) OR (intestinal parasite)) OR (protozoa)) OR (*Entamoeba coli*)) OR (*Giardia*)) OR (*Entamoeba histolytica*)) OR (*Cryptosporidium*)) OR (*Ascaris*)) OR (*Trichuris*)) OR (*Ancylostoma*)) OR (*Necator*)) OR (hookworm)) OR (whipworm)) OR (roundworm)) OR (pinworm)) OR (deworm)) OR (*Strongyloides*) AND (Humans)
EMBASE	#1 ‘body fat’ OR ‘bmi’ OR ‘body composition’ OR overweight OR obesity
	#2 ‘parasitic infections’ OR parasites OR helminth OR ‘intestinal parasite’ OR protozoa OR ‘*Entamoeba coli*’ OR *Giardia* OR ‘*Entamoeba histolytica*’ OR ‘*Cryptosporidium*’ OR ‘*Ascaris*’ OR ‘*Trichuris*’ OR ‘*Ancylostoma*’ OR ‘*Necator*’ OR ‘hookworm’ OR ‘whipworm’ OR ‘roundworm OR ‘pinworm’ OR ‘deworm’ OR ‘*Strongyloides*’
	#3 humans
	#4 (combination)—#1 AND #2 AND #3
Cochrane Library	#1 body fat
	#2 BMI
	#3 body composition
	#4 overweight
	#5 obesity
	#6 (combination)—#1 OR #2 OR #3 OR #4 OR #5
	#7 parasitic infections
	#8 parasites
	#9 helminth
	#10 intestinal parasite
	#11 protozoa
	#12 *Entamoeba coli*
	#13 *Giardia*
	#14 *Entamoeba histolytica*
	#15 *Cryptosporidium*
	#16 *Ascaris*
	#17 *Trichuris*
	#18 *Ancylostoma*
	#19 *Necator*
	#20 hookworm
	#21 whipworm
	#22 roundworm
	#23 pinworm
	#24 deworm
	#25 *Strongyloides*
	#26 (combination)—#7 OR #8 OR #9 OR #10 OR #11 OR #12 OR #13 OR #14 OR #15 OR #16 OR #17 OR #18 OR #19 OR #20 OR #21 OR #22 OR #23 OR #24 OR #25
	#27—Humans
	#28 (combination)—#6 AND #26 AND #27

**Table 3:** Characteristics of the experimental studies included (RCT’s).

Author, Year	Study Setting, Year (s)	No. of Participants	Age of the Participants	Intestinal Parasite Infections (IPI’s) Reported	Body Composition Reported	Treatment	Follow Up	Major Outcome
Stephenson et al., 1989	Kenya, 1986	150	6–16 y	*Ascaris*, Hookworm, *Trichuris*	WH, Triceps, Subscapular SF	Albendazole	6 mo	i. The ALB group showed a larger increase in percentage of WH (4.3 percentage points) with a *p*-value < 0.0002; ii. The ALB group showed a larger increase in triceps SF (1.2 mm more) with a *p*-value < 0.0002; iii. The ALB group showed a larger increase in subscapular SF (1.2 mm more) with a *p*-value < 0.0002.
Wiria et al., 2013a	Indonesia, 2008–2010	4004	25.7 ± 18.7 y	*Ascaris*, Hookworm, *Trichuris*, *Strongyloides*, *Ancylostoma*	BMI	Albendazole	9 mo, 21 mo	No significant change in BMI in children (9 mo follow-up: BMIZ: −0.04 (−0.17–0.09); 21 mo follow-up: BMIZ: −0.07 (−0.23–0.10)) or in adults (9 mo follow-up: BMI: −0.10 (−0.29–0.09); 21 mo follow-up: BMI: −0.15 (−0.29–0.10)) after Albendazole treatment.

ALB group: Albendazole group; BMI: Body Mass Index; BMIZ: Body Mass Index z-score; mo: months; SF: skinfolds; RCT’s: Randomized Controlled Trials; y: years old; WH: Weight-for-height.

**Table 4:** Association between intestinal parasite infections and direct measures of body composition.

Author, Year	Age Range	Body Composition	Intestinal Parasitic Infection	MD ^I vs. U^	RR	OR	95% CI	*p*-Value
Zavala et al., 2016	6–10 y	Body fat (%)	IPI	−0.3	NR	NR	NR	0.968
	6–10 y	Abdominal fat (%)	IPI	−0.2	NR	NR	NR	0.919
	6–10 y	Body fat (kg)	IPI	0.2	NR	NR	NR	0.873
	6–10 y	Abdominal fat (kg)	IPI	0	NR	NR	NR	0.636
Jardim-Botelho et al., 2008	<18 y	Low lean mass	*Ascaris*	NR	NR	1.93	1.00–2.16	0.048
	<18 y	Low fat mass	*Ascaris*	NR	NR	1.68	1.12–2.51	0.12
	>18 y	Low fat mass	*Ascaris*	NR	NR	1.87	1.07–3.28	0.029
	>18 y	Low fat mass	Hookworm	NR	NR	1.91	1.08–3.35	0.025
Gerber et al., 2018	8–12 y	Body composition	*Ascaris*	NR	0.98	NR	0.92–1.04	NR
	8–12 y	Body composition	*Trichuris*	NR	0.93	NR	0.86–1.01	NR
	8–12 y	Body composition	*Ascaris* or *Trichuris*	NR	0.93	NR	0.86–1.02	NR

IPI: Intestinal Parasite Infections (helminth and protozoa); I vs. U: Infected versus Uninfected; MD: Mean Difference; NR: Not Reported; OR: odd ratio (adjusted); RR: Relative Risk; y: years-old; 95% CI: Confidence Interval. Low lean mass, Low fat mass, Body and Abdominal fat (% and kg) were all determined by DEXA.

**Table 5:** Associations between intestinal parasite infection status and proxy (weight-for-height) measures of body composition.

Author, Year	Age Range	Body Composition	Intestinal Parasite	MD ^I vs. U^	OR	β	95% CI	*p*-Value
Amare et al., 2013	12.09 ± 2.54 y	BMIZ (♀)	IPI	−0.36 ^c^	NR	NR	NR	0.3
	12.09 ± 2.54 y	BMIZ (♂)	IPI	−0.19 ^c^	NR	NR	NR	0.143
Orden et al., 2014	3–11 y	BMIZ (Berisso)	IPI	−0.3	NR	NR	NR	0.138
	3–11 y	BMIZ (Magdallena)	IPI	0.00	NR	NR	NR	0.969
Sayasone et al., 2015	6 mo–12 y	<−2 BMIZ	2 Helminths	NR	1.40	NR	1.01–1.94	NR
Sanchez et al., 2013	7–14 y	BMIZ	>3 Helminths	NR	1.45	NR	1.03–2.04	NR
			Helminths	NR	NR	−0.06	−0.31–0.19	0.622
Verhagen et al., 2013	4–16 y	BMIZ or −WHZ ^1^	Helminths	NR	NR	−0.24	−0.46–−0.01	NR
Quihui-Cota et al., 2015	6–13 y	BMIZ	*Cryptosporidium*	0.05 ^c^	NR	NR	NR	0.68
Zavala et al., 2016	6–10 y	BMIZ	IPI	0.20 ^c^	NR	NR	NR	0.283
Zavala et al., 2019	1–5 y	BMIZ	*Ascaris*	NR	NR	−0.32	−0.33–−0.31	<0.01
	6–10 y	BMIZ	*Ascaris*	NR	NR	−0.21	−0.22–0.19	0.01
	6–10 y	BMIZ	Protozoa	NR	NR	0.61	0.59–0.63	<0.01
	11–18 y	BMIZ	Protozoa	NR	NR	0.85	0.83–0.88	<0.01
Zhou et al., 2007	10–12 y	BMIZ	*T. trichiura*	NR	NR	−0.22	NR	0.001
Patel and Khandekar, 2006	9–10 y	BMI	IPI	−0.46	NR	NR	NR	>0.05
Li et al., 2015	50–70 y	BMI (≤18 vs. >18)	Protozoa	NR	3.3	NR	1.44–7.54	NR
	50–70 y	BMI (≤18 vs. >18)	Helminths	NR	3.32	NR	1.39–7.91	NR
Wiria et al., 2013b	18–80 y	BMI	Helminths	−0.66	NR	NR	−1.26–−0.06	0.031
Gerber et al., 2018	8–12 y	BMI	*Ascaris*	NR	0.91 ^7^	NR	0.83–1.00	NR
	8–12 y	BMI	*Trichuris*	NR	0.89 ^7^	NR	0.89–0.99	NR
Kurscheid et al., 2020	8–12 y 2–12 y	BMI BMI	*Ascaris* + *Trichuris* *Ascaris* and/or *Trichuris* and/or Hookworm	NR 0.1 ^c^	0.90 ^7^ NR	NR NR	0.82–0.99 NR	NR 0.79
Casapia et al., 2007	<5 y	WHZ < 0.2 SD	*T. trichiuria*	NR	2.5	NR	1.06–5.93	NR
	<5 y	WHZ < 0.2 SD	Hookworm	NR	6.67	NR	1.08–41.05	NR
	<5 y	WHZ < 0.2 SD	*A. lumbricoides*	NR	3.577	NR	1.79–7.09	NR
Campos-Ponce et al., 2012	0–19 y, >19 y	Overweight/obese ^2^	IPI	NR	1.47	NR	0.51–4.27	0.48
Geltman et al., 2001	<18 y	Overweight/obese ^3^	IPI	NR	0.5	NR	0.20–0.80	NR
Rivero et al., 2018	<15 y	Overweight/obese ^4^	STH	NR	NR	0.752	NR	0.33
	<15 y	Overweight/obese ^4^	*G. duodenalis*	NR	−1.053 ^8^	NR	NR	0.016
Chu et al., 2013	7–14 y	Overweight ^5^	Pinworm	NR	0.45	NR	0.28–0.71	<0.01
	7–14 y	Obese ^6^	Pinworm	NR	0.42	NR	0.26–0.70	<0.01
Lander et al., 2015	3–6 y	BMIZ	Helminth	NR	NR	−0.004 ^9^	NR	<0.01
Wasilewska et al., 2011	1–18 y	<1 BMIZ	*Ascaris*	NR	0.98	NR	0.28–3.46	0.974

♀ Girls, ♂ Boys. BMI: Body Mass Index; I vs. U: Infected versus Uninfected; ^c^: Calculated by authors; BMIZ: BMI for age z-score; WHZ: Weight-for-Height z-score; IPI: Intestinal Parasite Infections (not specified); MD: Mean Difference, NR: Not Reported; OR: Odd Ratio (adjusted); RR (relative risk); β: coefficient of linear regression; 95% CI: Confidence Interval; y: years old; SD: standard deviation; ^1^: Weight-for-Height z-score in children < 5 years (*n* = 37) and BMI-for-age z-score in children ≥ 5 years (*n* = 338); ^2^: Overweight/obese defined per age range: 0–5 y: BMI for age > 2 SD; 5–19 y: BMI for age > 1 SD; >19 y: BMI for age > 25 SD; ^3^: Overweight/obese defined as: BMI > 84th percentile; ^4^: Overweight/obese defined as: +1 < BMIZ < +2 for overweight and BMIZ > +2 for obese; ^5^ Overweight defined as: BMI > 85th percentile; ^6^: Obese defined as BMI ≥ 95th percentile. ^7^ Relative Risk have been reported; ^8^ log(OR) has been reported; ^9^ Direct effect using the standardized regression weight from the structural equations model.

## Data Availability

The data presented in this study are available on request from the corresponding author. The data are not publicly available due to the ethical reasons.

## Supplementary Materials

The following supporting information can be downloaded at: https://www.mdpi.com/article/10.3390/nu14112229/s1, Table S1: Preferred Reporting Items for Systematic reviews and Meta-Analyses extension for Scoping Reviews (PRISMA-ScR) Checklist and Table S2: Study design and quality assessment of the studies included in scoping review.
