# Supplementary material for: Association between Intestinal Parasite Infections and Proxies for Body Composition: A Scoping Review
Source: Nutrients. 2022 May 26;14(11):2229. doi: 10.3390/nu14112229 (PMC9182792; doi:10.3390/nu14112229)
Supplement: Supplementary file 1 [file nutrients-14-02229-s001.zip › Table S2 - Checklist JBI.pdf]

Table S2: **Study design and quality assessment of the studies included in scoping review.**

| Nr | Author, Year | Q1 | Q2 | Q3 | Q4 | Q5 | Q6 | Q7 | Q8 | Q9 | Q10 | Q11 | Q12 | Q13 | Quality score |
|---|---|---|---|---|---|---|---|---|---|---|---|---|---|---|---|
| 1 | Amare *et al.*, 2013 | Y | Y | Y | Y | Y | Y | Y | Y | | | | | | 100% |
| 2 | Campos-Ponce *et al.*, 2012 | Y | Y | Y | Y | Y | Y | Y | Y | | | | | | 100% |
| 3 | Casapia *et al.*, 2007 | Y | Y | Y | Y | Y | Y | Y | Y | | | | | | 100% |
| 4 | Chu *et al.*, 2013 | Y | Y | Y | Y | Y | Y | Y | Y | | | | | | 100% |
| 5 | Geltman *et al.*, 2001 | Y | Y | Y | Y | Y | Y | Y | Y | | | | | | 100% |
| 6 | Gerber *et al*, 2018 | Y | Y | Y | Y | Y | Y | Y | Y | | | | | | 100% |
| 7 | Jardim-Botelho *et al.*, 2008 | Y | Y | Y | Y | Y | Y | Y | Y | | | | | | 100% |
| 8 | Kurscheid *et al*, 2020 | Y | Y | Y | Y | Y | Y | Y | Y | | | | | | 100% |
| 9 | Lander *et al.*, 2015 | Y | Y | Y | Y | Y | Y | Y | Y | | | | | | 100% |
| 10 | Li *et al.*, 2015 | Y | Y | Y | Y | Y | Y | Y | Y | | | | | | 100% |
| 11 | Orden *et al.*, 2014 | Y | Y | Y | Y | Y | Y | Y | Y | | | | | | 100% |
| 12 | Patel & Khandekar, 2006 | Y | Y | Y | Y | Y | Y | Y | Y | | | | | | 100% |
| 13 | Rivero *et al.*, 2018 | Y | Y | Y | Y | Y | Y | Y | Y | | | | | | 100% |
| 14 | Quihui-Cota *et al.*, 2015 | Y | Y | Y | Y | Y | Y | Y | Y | | | | | | 100% |
| 15 | Sanchez *et al.*, 2013 | Y | Y | Y | Y | Y | Y | Y | Y | | | | | | 100% |
| 16 | Sayasone *et al.*, 2015 | Y | Y | Y | Y | Y | Y | Y | Y | | | | | | 100% |
| 17 | Stephenson *et al.*, 1989* | Y | Y | Y | Y | Y | Y | N | Y | Y | Y | Y | Y | Y | 92% |
| 18 | Verhagen *et al.*, 2013 | Y | Y | Y | Y | Y | Y | Y | Y | | | | | | 100% |
| 19 | Wasilewska *et al.*, 2011 | Y | Y | Y | Y | Y | Y | Y | Y | | | | | | 100% |
| 20 | Wiria *et al.*, 2013a* | Y | Y | Y | Y | Y | Y | Y | Y | Y | Y | Y | Y | Y | 100% |
| 21 | Wiria *et al.*, 2013b | Y | Y | Y | Y | Y | Y | Y | Y | | | | | | 100% |
| 22 | Zavala *et al.*, 2016 | Y | Y | Y | Y | Y | Y | Y | Y | | | | | | 100% |
| 23 | Zavala *et al.*, 2019 | Y | Y | Y | Y | Y | Y | Y | Y | | | | | | 100% |
| 24 | Zhou *et al.*, 2007 | Y | Y | Y | Y | Y | Y | Y | Y | | | | | | 100% |

* RCTs

**Key: Y**= Yes; **N**= Not reported

**Question codes:**

**Cross-sectional**

1. Were the criteria for inclusion in the sample clearly defined?

2. Were the study subjects and the setting described in detail?

3. Was the exposure measured in a valid and reliable way?

4. Were objective, standard criteria used for measurement of the condition?

5. Were confounding factors identified?

6. Were strategies to deal with confounding factors stated?

7. Were the outcomes measured in a valid and reliable way?

8. Was appropriate statistical analysis used?

**RCTs**

1. Was true randomization used for assignment of participants to treatment groups?

2. Was allocation to treatment groups concealed?

3. Were treatment groups similar at the baseline?

4. Were participants blind to treatment assignment?

5. Were those delivering treatment blind to treatment assignment?

6. Were outcomes assessors blind to treatment assignment?

7. Were treatment groups treated identically other than the intervention of interest?

8. Was follow up complete and if not, were differences between groups in terms of their follow up adequately described and analyzed?

9. Were participants analyzed in the groups to which they were randomized?

10. Were outcomes measured in the same way for treatment groups?

11. Were outcomes measured in a reliable way?

12. Was appropriate statistical analysis used?

13. Was the trial design appropriate and any deviations from the standard RCT design (individual randomization, parallel groups) accounted for in the conduct and analysis of the trial?
